# DoMY-Seq: A yeast two-hybrid–based technique for precision mapping of protein–protein interaction motifs

**DOI:** 10.1074/jbc.RA120.014284

**Published:** 2020-11-23

**Authors:** Pau Castel, Ann Holtz-Morris, Yongwon Kwon, Bernhard P. Suter, Frank McCormick

**Affiliations:** 1 Helen Diller Family Comprehensive Cancer Center, University of California San Francisco, San Francisco, California, USA; 2 Next Interactions, Richmond, California, USA

**Keywords:** yeast two-hybrid, protein–protein interaction, next-generation sequencing, domains, cDNA, complimentary DNA, CRD, cysteine-rich domain, CRISPR, clustered regularly interspaced short palindromic repeats, DoMY-Seq, Protein Domain mapping using Yeast 2 Hybrid-Next Generation Sequencing, GTP, guanosine triphosphate, KRAS, Kirsten rat sarcoma, MDM2, mouse double minute 2 homolog, MEK, Mitogen-Activated Protein Kinase/Extracellular Signal–Regulated Kinase Kinase, NGS, next-generation sequencing, ORF, open reading frame, p53, tumor protein p53, PPI, protein–protein interaction, RBD, Ras-binding domain, RGL3, Ral GEF-like 3, RIT1, Ras-like without CAAX 1, Y2H, yeast two-hybrid

## Abstract

Interactions between proteins are fundamental for every biological process and especially important in cell signaling pathways. Biochemical techniques that evaluate these protein–protein interactions (PPIs), such as *in vitro* pull downs and coimmunoprecipitations, have become popular in most laboratories and are essential to identify and validate novel protein binding partners. Most PPIs occur through small domains or motifs, which are challenging and laborious to map by using standard biochemical approaches because they generally require the cloning of several truncation mutants. Moreover, these classical methodologies provide limited resolution of the interacting interface. Here, we describe the development of an alternative technique to overcome these limitations termed “Protein Domain mapping using Yeast 2 Hybrid-Next Generation Sequencing” (DoMY-Seq), which leverages both yeast two-hybrid and next-generation sequencing techniques. In brief, our approach involves creating a library of fragments derived from an open reading frame of interest and enriching for the interacting fragments using a yeast two-hybrid reporter system. Next-generation sequencing is then subsequently employed to read and map the sequence of the interacting fragment, yielding a high-resolution plot of the binding interface. We optimized DoMY-Seq by taking advantage of the well-described and high-affinity interaction between KRAS and CRAF, and we provide high-resolution domain mapping on this and other protein-interacting pairs, including CRAF-MEK1, RIT1-RGL3, and p53-MDM2. Thus, DoMY-Seq provides an unbiased alternative method to rapidly identify the domains involved in PPIs by advancing the use of yeast two-hybrid technology.

PPIs are defined as the physical contact between two or more proteins. PPIs are mediated by either covalent or noncovalent bonds and can also be transient or stable, depending on the biochemical and biophysical characteristics of the interaction. Several classes of chemical bonds are known to participate in PPIs and include, but are not limited to, hydrogen, ionic, hydrophobic, disulfide bonds, and Van der Waals forces (1, 2).

Such PPIs are necessary for virtually all biological processes, mediating, for example, the recognition of substrates during signal transduction reactions, the engagement between receptors and ligands, host–pathogen interactions, the regulation of metabolism, proteolysis, the epigenetic code, and the nucleic acid mechanisms, among many others (3). PPIs are important not only in the normal physiology but also in pathophysiology, where either loss or gain of these interactions can lead to the diseased phenotype (4). For instance, some oncoproteins, such as KRAS, are known to have increased binding affinity for effector proteins that are able to propagate the downstream signal that leads to proliferation and survival (5).

Elucidating PPI motifs is critical for characterizing novel interactions and can be used to define unique domains, generate loss-of-function mutations, or assist structural and drug discovery studies. Given the increasing interest and development of small-molecule therapeutics that disrupt PPIs, methodologies that improve the speed, quality, and resolution of PPI motifs are highly sought after (6).

Several techniques have been established to study the interactions between two proteins, the most commonly used being pull-down assays, coimmunoprecipitation, yeast two-hybrid (Y2H) technique, fluorescence-based assays, and mass spectrometry-based approaches (7). For PPI motif identification, the use of randomly truncated forms of the protein of interest is widely employed. These fragments are generated based on domains predicted bioinformatically or arbitrarily within the protein, giving rise to either overlapping or nonoverlapping fragments that are tagged and used for *in vitro* pull down or coimmunoprecipitation in cultured cells (8). This method has several disadvantages, including the low-throughput and laborious process of cloning and expressing such fragments, the biased approach when designing the fragments, and the low resolution of the assay, given the difficulty of assessing fragments shorter than 5 kDa. Moreover, the expression of the fragments is generally unequal, and the arbitrary cuts can interfere with the PPI interphase. Hence, when a PPI motif is identified, follow-up structural studies are highly desirable to improve the resolution of the minimal PPI motif. To overcome these technical and conceptual challenges, we have developed “Protein Domain mapping using Yeast 2 Hybrid-Next Generation Sequencing” (DoMY-Seq), an approach that uses Y2H technology to identify PPI motifs by means of next-generation sequencing (NGS). Our procedure provides a rapid, unbiased, and reliable platform to determine the interacting motif of a specific known interactor.

## Results

### Design and overview of DoMY-Seq

**Figure 1:**
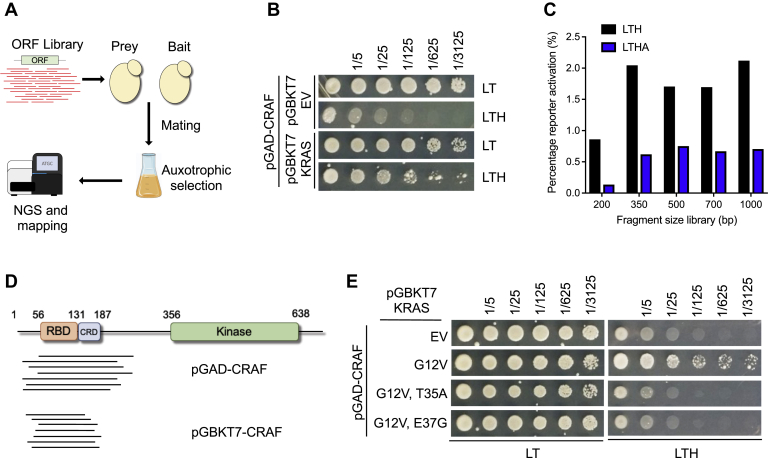
**Overview and optimization of DoMY-Seq pipeline.***A*, using a fragment library obtained from the open reading frame (ORF) of a known interactor, DoMY-Seq allows mapping of the interaction motifs upon yeast mating, auxotrophic selection, and next-generation sequencing (NGS). *B*, representative colonies obtained between the KRAS bait and a CRAF prey library using Y2H. LT (-Leu/-Trp) and LTH (-Leu/-Trp/-His) indicate auxotrophic media used for selection. Cultures were diluted (1/5–1/3125) as indicated. *C*, percentage of Y2H reporter activation using different fragment size libraries of CRAF against the KRAS bait. Higher stringency selection was achieved using the LTHA (-Leu/-Trp/-His/-Adenine) auxotrophic selection media. *D*, representative Sanger sequencing fragments from colonies obtained from different bait/prey orientations. *E*, effect of the KRAS effector–binding domain mutations T35A and E37G in the interaction of CRAF prey using Y2H. Cultures were diluted (1/5–1/3125) as indicated. DoMY-Seq, Protein Domain mapping using Yeast 2 Hybrid-Next Generation Sequencing.

In order to develop a novel technique suitable for the rapid and unbiased discovery of high-resolution PPI motifs, we took advantage of the Y2H technology, which traditionally allows for the qualitative determination of PPIs with a specific bait protein using a high-content library of potential preys (9, 10). We complemented this approach by generating a Y2H prey library that contained thousands of random fragments derived from the open reading frame (ORF) of an interactor of interest. This was achieved by shearing and subcloning DNA fragments from a reference plasmid that encoded the interactor of interest using standard library preparation protocols (See Experimental procedures and Fig. S1). Next, the fragment library, following cloning into the auxotrophic binary system, was combined with yeast of the opposite mating type containing the bait and selected for diploid yeast cells that exhibited Y2H reporter activation with the appropriate auxotrophic factors. After the selection process, DNA was isolated from the enriched cell populations and prey library compositions in these populations were analyzed with an Illumina NGS protocol. To define the motifs in the prey library that bound to the bait protein, DNA reads were aligned to the reference plasmid. The exact location of the PPI motif was then determined at single amino-acid resolution by fragment overlap analysis from a saturating amount of prey library fragments (Fig. 1*A*).

### Generation and optimization of PPI motif libraries

To develop a reliable and optimized method for generation of a library from a plasmid encoding for a protein of interest, we used the interacting partners KRAS (the 4A splice isoform) and CRAF, as a prototype PPI. In fact, the interaction between these two proteins was first described in a Y2H screening and largely occurs through interaction of the N-terminal (aa 56–131) Ras-binding domain (RBD) of CRAF and the effector-binding domain of KRAS (aa 32–40) in a GTP-dependent manner (11, 12). To facilitate this interaction, we started by using the KRAS mutant G12V, which exhibits high levels of loaded GTP in eukaryotic cells (5, 13) and confirmed the interaction of KRAS G12V bait protein when mated with yeast carrying the plasmid encoding full-length CRAF in the prey vector (Fig. 1*B*). Next, based on the compatibility of the system, we generated prey fragment libraries using a plasmid encoding full-length CRAF as a template. In order to establish the most appropriate fragment library length for our assays, we produced fragments of approximately 1000 bp, 700 bp, 500 bp, 350 bp, and 200 bp, to use as prey against our KRAS bait (Fig. 1*C*). The fragment size did not have an important impact on the number of positive colonies selected in auxotrophic media, which represents the degree of interaction between the fragments and our bait. Only the libraries containing 200-bp fragments yielded a lower number of colonies than the libraries containing longer fragments. For novel interactors, we recommend testing the length of the library prior to proceeding with the assay and avoid libraries containing short fragments (<200 bp). Given that the 350- and 500-bp fragments were expected to encode 100- to 150-aa fragments and that the interacting motif of CRAF (RBD) is ∼75 aa, we decided to select these conditions for further analysis.

We also tested whether the orientation of the bait and prey was important for the assay. We swapped the CRAF (pGAD) and KRAS (pGBKT7) ORFs into the pGBKT7 and pGAD plasmids, respectively. Given that no differences in colony formation were detected, we picked several colonies from the direct (pGAD-CRAF) and reverse assay (pGBKT7-CRAF) for Sanger sequencing to confirm the identity of the interactors. For both assays, we could align these in-frame sequences to the CRAF RBD, suggesting that the orientation of the bait and prey did not interfere with the assay (Fig. 1*D*). To further address the specificity of the interaction toward our bait, we introduced two separate point mutations in the effector domain of KRAS, namely T35A and E37G substitutions, that render KRAS unable to bind most protein effectors (14). In our colony formation assays, we found that these mutants are unable to produce interacting colonies, confirming the specificity of the interaction (Fig. 1*E*).

### Optimization of the KRAS bait

Similar to our prey library, we questioned whether certain conditions could have an impact on the performance of our bait. Because KRAS requires bound GTP to interact with CRAF and different oncogenic mutations of KRAS have been shown to be associated with different levels of GTP (15), we addressed whether distinct mutations could yield a higher number of colonies. Our colony formation assays did not show differences between WT, G12V, G12C, G12D, G13D, and Q61L mutants, suggesting that when KRAS is expressed in the yeast cells, the levels of GTP are higher than those in mammalian cells (Fig. S2*A*), as reported previously (13).

KRAS cellular trafficking can also have an impact on the interaction with RAF paralogs, and nuclear localization is important for reporter activation in the Y2H assay. Next, we addressed whether the localization of our bait could result in improved yields of reporter activation by generating point mutations at the KRAS prenylation sites C180 and C186 (16). The C186S mutation at the CAAX box, which abrogates farnesylation of KRAS, produced a higher number of colonies selected in auxotrophic media (Fig. S2*B*). This result is in line with that of previous Y2H experiments in which the KRAS bait only encodes for the G domain, thereby increasing the localization in the nuclei, where reporter activation takes place (17). Despite the improved reporter activation, we did not find sufficient advantages for using this variant.

We also observed some background activation with the pGBKT7 empty vector control in these panels. Since the DBD in pGBKT7 is expressed at a very high level, this likely reflects nonspecific reporter activation. However, this background is not consistently observed (see Fig. 1, *B* and *E*), and it does not interfere with the mapping assays. Nevertheless, when undertaking DoMY-Seq experiments, it is generally recommended to test the level of background with empty vector control, especially upon prolonged selective growth, to avoid potential low signal-to-noise ratio.

### High-throughput sequencing enables interacting domain mapping of KRAS binding to CRAF RBD

**Figure 2:**
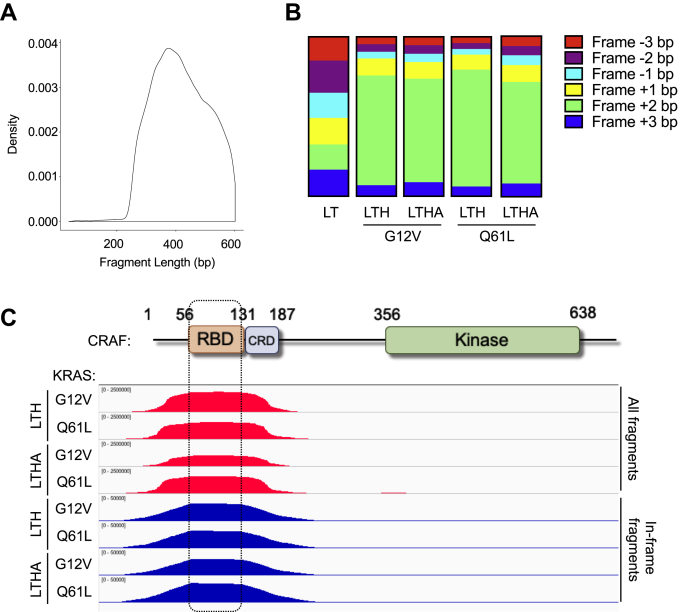
**DoMY-Seq allows mapping of the KRAS-binding domain in CRAF.***A*, distribution of the fragment length obtained in the CRAF library. *B*, percentage of CRAF fragments classified by their reading frame. Frame +2 bp (*green*) represents the coding (in-frame) fragments. Note the selection of the coding fragments in the presence of the auxotrophic dropout media (LTH and LTHA), but not in the basal media (LT) in which fragments are distributed uniformly. *C*, integrative Genomics Viewer (IGV) snapshots of the CRAF peaks obtained with DoMY-Seq depicting domain interaction with KRAS (G12V or Q61L) baits. Results from the both LTH and LTHA dropout media are showed. DoMY-Seq, Protein Domain mapping using Yeast 2 Hybrid-Next Generation Sequencing.

We performed high-throughput sequencing by amplifying the specific adaptors present in the prey fragment library that was selected in low- and high-stringency media (LTH [-Leu/-Trp/-His] and LTHA [-Leu/-Trp/-His/-Adenine], respectively). Upon analysis, we found that in all conditions tested, the average length of the fragments selected was between 300 and 400 bp, consistent with the initial fragment size of our libraries (Fig. 2*A*). As a result of random fragmentation during library preparation, six reading frames can be expected, designated as −3 bp, −2 bp, −1 bp, +1 bp, +2 bp, and +3 bp. While most of these fragments are out of frame and do not encode for any identifiable peptide before selection, the in-frame (designated as +2 bp) fragments encode for the expected CRAF peptides to be mapped upon selection.

We examined the distribution of the six frames within our library before and after auxotrophic media selection. The distribution of the six frames was similar in our preselection media control library ($\bar{\text{x}}$ = 16.67 ± 0.8%) but was highly enriched for the in-frame fragments (+2 bp) when selected with either LTH or LTHA media (Fig. 2*B*). This indicates that when the prey–bait interaction takes place, thereby activating the prototrophic reporter, it occurs mainly through the fragments that encode for CRAF. We used the data obtained from our experiments to align the sequences against the CRAF reference sequence (“peak calling”) and found a clear enrichment in the region encoding for the RBD and the CRD domain. When only in-frame fragments were considered for the analysis, the peak of our coverage plot was strictly limited to the region between aa 58 to 128 (70 aa), where the RBD is located (Fig. 2*C*). Moreover, some coverage was also observed in the adjacent sequence encoding for the CRD domain, suggesting the existence of putative additional PPI contacts between this domain and KRAS, as previously suggested by biochemical experiments (18). The exact same minimal binding domain for CRAF binding was also mapped for oncogenic KRAS variant Q61L (Fig. 2*C*). Moreover, the exact match to positions 58 to 128 was also observed when KRAS G12V binding was assayed with a different library of longer CRAF fragments with ∼500-bp fragment insert length or when the library was cloned in the reversed plasmid orientation (Fig. S3). Hence, interaction site mapping *via* DoMY-Seq using saturating amounts of DNA fragments delivers precise and highly reproducible results.

### DoMY-Seq identifies MEK1 kinase–interacting interface in CRAF

**Figure 3:**
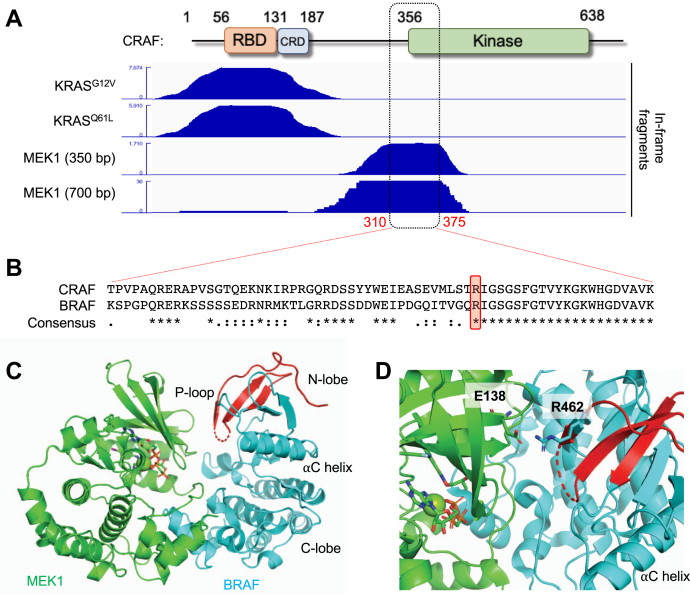
**DoMY-Seq reveals the interaction interface between CRAF and MEK1.***A*, integrative Genomics Viewer (IGV) snapshots highlighting the CRAF-interacting domains of KRAS and MEK1 baits. The MEK1-binding motif is defined within aa 310 to 375. Note that shorter fragments (350 bp) provide better resolution than larger fragments (700 bp) in this assay. For each assay, two biological independent assays were performed. *B*, alignment between CRAF and BRAF shows a number of conserved amino acids present in the 310 to 375 motif. CRAF R354 and BRAF R462 are highlighted in *red*. *C*, overall structure of the BRAF (*cyan*) and MEK1 (*green*) interaction (PBD: 4MNE). Regulatory motifs of the BRAF kinase domain have been labeled. The aligned region of the CRAF MEK1 binding motif (310–375) identified by DoMY-Seq is highlighted in *red* and is present within the BRAF P-loop. *D*, a detailed snapshot of (*C*) reveals the presence of the key interacting residues R462 (BRAF) and E138 (MEK1). BRAF R462 is conserved in CRAF (R354). DoMY-Seq, Protein Domain mapping using Yeast 2 Hybrid-Next Generation Sequencing.

Using the same experimental approach, we leveraged the ∼350-bp CRAF fragment library to identify the core binding site of MEK1, a downstream kinase that is phosphorylated by the RAF paralogs ARAF, BRAF, and CRAF (19). DoMY-Seq revealed a peak at the N terminus of the CRAF kinase domain (Fig. 3*A*), which included the precise MEK1-binding sequence from aa 310 to 375 (65 aa). Interestingly, coverage of the sequences adjacent to the core binding site or peak was very asymmetrical, with more fragments overlapping into the intradomain region 5’ from the binding site, while fragment coverage stopped sharply on the 3’ side. This could suggest the preferential association with this region or the presence of an adjacent amino acid sequence in the kinase domain region that precludes the binding of MEK1. We also used the 700-bp CRAF fragment library to map the MEK1-binding site and found that the longer fragments yielded a slightly larger binding site on the 5’ site, while the 3’ site remained similar, supporting the idea that this downstream sequence could provide a negative binding effect.

BRAF is a paralog of CRAF (47% sequence identity) that is highly mutated in malignancies such as melanoma, thyroid carcinoma, colorectal cancer, and hairy cell leukemia, as well as the rare disease non–Langerhans cell histiocytosis (20). Given the interest in BRAF as a drug target, structural data have been reported for this kinase in the apo form or in complex with small molecules, another RAF protomer, MEK1, or 14-3-3 (21, 22, 23, 24). Therefore, we examined whether the CRAF-binding site was conserved in the previously described cocrystal structure of BRAF/MEK1 (21). Residues 418 to 483 of BRAF align with residues 310 to 375 in CRAF (Fig. 3*B*). The amino acid sequence between BRAF R462 and K483 is identical to that between CRAF R354 and K375. At the structural level, this region is found at the P-loop of the BRAF kinase domain N-lobe (Fig. 3*C*). This region has been previously shown to act as an interface for MEK1 binding and mutations in the P-loop (*e.g.*, R462E) disrupt such interaction (21). A closer look suggests that R462 interacts with MEK1 E138 (Fig. 3*D*). While the BRAF-MEK1 crystal structure shows a secondary binding interface mediated by BRAF residues I617, N661, and I666, we did not detect the corresponding CRAF sequence by DoMY-Seq. This could be because the interaction between CRAF and MEK1 is different than that between BRAF and MEK1 or because DoMY-Seq failed to capture that second binding motif.

### DoMY-Seq defines the interactions between RIT1 GTPase and RGL3

**Figure 4:**
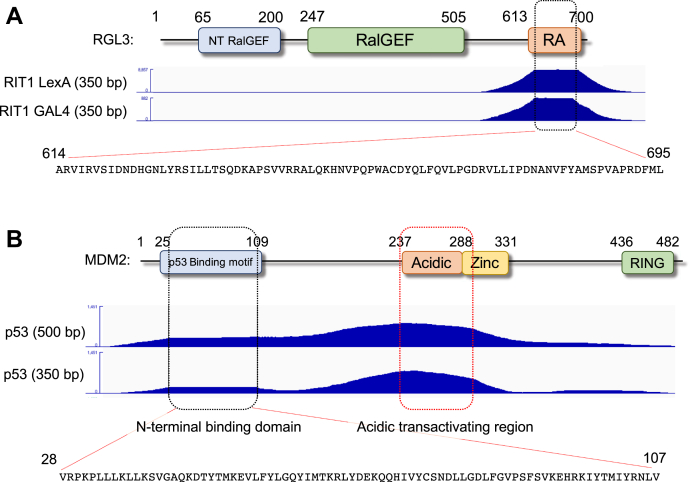
**Identification of RIT1-RGL3– and p53-MDM2–binding domains by DoMY-Seq.***A*, integrative Genomics Viewer (IGV) snapshots of the RIT1-interacting domain in RGL3. Note that by using two different bait vectors and reporter systems, we obtained the same interaction motif, which is depicted below and maps within the RGL3 Ras association (RA) domain. For each assay, two biological independent assays were performed. *B*, similar snapshots depict the p53-binding motifs of MDM2 using two fragment size prey libraries. We observed two peaks that map to the canonical N-terminal p53 binding domain and the acidic transactivating region. Given the presence of acidic 9-aa TAD sequences, this motif can indicate a previously described binding domain or a false-positive transactivating sequence. For each assay, two biological independent assays were performed. DoMY-Seq, Protein Domain mapping using Yeast 2 Hybrid-Next Generation Sequencing; MDM2, mouse double minute 2 homolog; TAD, transactivation domain.

Next, we asked whether DoMY-Seq could also be used to identify the interaction interface between other GTPases and effector proteins containing Ras-binding domains. For this purpose, we used RIT1, a small GTPase from the Ras family that has recently been involved in the pathogenesis of Noonan syndrome, a neurodevelopmental disorder (25). Several protein effectors have been identified for RIT1, but for RGL3, a Ral GTPase exchange factor, the exact binding motif has not been previously mapped at the amino acid resolution (26). Using the LexA system, the RIT1–RGL3 interaction yielded a significant number of positive colonies. Therefore, we generated a prey library containing 350-bp RGL3 fragments and tested their ability to interact with RIT1 (Fig. 4*A*). DoMY-Seq revealed an interaction peak that mapped between amino acid positions 614 to 695 (81 aa), consistent with the presence of a homology-based annotated Ras-association domain (RA domain, amino acid positions 613–700 in RGL3). This predicted domain has been shown in biochemical assays to bind to the GTP-loaded form of RIT1 (27). When the assay was repeated using the Gal4-based Y2H system, we observed the same overlapping sequence, highlighting the consistency of our assay when using different reporter systems.

### Mapping of the p53-binding site in mouse double minute 2 homolog (MDM2) E3 ligase

Lastly, we applied DoMY-Seq to characterize the binding site between the p53 tumor suppressor protein and the human MDM2 E3 ubiquitin ligase, which ubiquitinates p53 for proteasomal degradation (28). The p53–MDM2 interaction is an established therapeutic target, and a variety of small-molecule drugs and peptidomimetics that destabilize this interaction have been developed (29, 30). The interaction is also readily detectable in the Y2H assay, which can be exploited for drug targeting assays (31). The direct interaction between the two proteins has been localized to a relatively small (aa 25–109) hydrophobic pocket domain at the NH_2_ terminus of MDM2 and a 15-aa amphipathic peptide at the NH_2_ terminus of p53 using biochemical methods and crystallography (32, 33). This domain constitutes the minimal binding sequences required, although other contacts occur between the two proteins (34, 35). We generated 2 fragment libraries from MDM2, one with an average insert length of ∼350 aa and the other with ∼500 aa. Since the transactivation domain in the N terminus of the p53 protein overlaps with the MDM2-binding domain, we used full-length p53 as the prey partner and performed the fragment mapping for MDM2 in the bait orientation. Using DoMY-Seq, we could see enrichment in MDM2 with a plateau at the N terminus (between aa 28 and107, total 80-aa binding site), which corresponds well with the previously determined hydrophobic binding pocket (Fig. 4*B*). We can assume that this domain contains the binding determinant that has been shown before, since it maps to the same region. We also see a very strong enrichment of fragments in the middle domain of MDM2, corresponding to the acidic domain. However, there is no distinct plateau and we cannot determine whether this peak is the result of a specific binding motif previously described in the acidic domain (34) or the presence of self-activating peptides in that region. Because sequences containing acidic, hydrophobic, and aromatic amino acids have been previously shown to act as transactivators when fused to a DNA-binding domain (36), and hence can result in false positives in Y2H, we decided to look at the presence of such sequences in MDM2. Using the previously described 9-aa transactivation domain prediction tool (37), which can identify peptide sequences with physicochemical properties that result in potential DNA transactivation, we found a sequence between aa 274 and 282 (EVYQVTVYQ) that corresponds to the highest fragment enrichment in the MDM2 library. This sequence could be transactivating, because of the combination of acidic, hydrophobic, and aromatic residues, or transactivation could be caused by additional acidic sequences flanking this region. Therefore, the observed peak could not only be the result of specific binding but also be due to the presence of an acidic motif, highlighting one of the possible limitations of using this approach. We evaluated all of our DoMY-Seq results and found that most regulatory sequences found in commonly used plasmids do not contain such transactivators, with the exception of the spectinomycin resistance gene (aminoglycoside adenylyltransferase) (38) (Fig. S4).

## Discussion

We have described a novel, fast, and inexpensive adaptation of the Y2H principle that allows rapid and precise characterization of binding domains between interacting proteins. The conventional use of Y2H is the discovery and exploration of novel binary PPIs which define biological mechanisms and pathways (9). Hence, NGS readouts for Y2H assays that have been developed so far aimed at the increase of screening throughput (3, 39, 40, 41). Here, we offer an alternative application of Y2H coupled with NGS, which is the detailed mapping of PPI interfaces down to the amino acid level. With complete saturation of a gene template with random DNA fragments and high sequencing capacity, DoMY-Seq allows the identification of core binding sequences and motifs, which can usually be achieved only with protein cocrystallization. The core binding domains can be further characterized with site-specific mutagenesis and provide an initial approach in structural biology to determine suitable protein fragments for cocrystallization.

While Y2H has long been established as a discovery tool, a common perception is that Y2H assays are especially prone to false-positive results. Since DoMY-Seq is primarily focused on the precise characterization of PPIs that are already known or supported by evidence, false positives are not considered a major challenge. Nevertheless, we have also performed a detailed analysis of peptides that encode for self-activators in the Y2H system that lead to false-positive results, so they can be recognized and filtered out (see Fig. S4).

On the other hand, we have observed that certain PPIs that have been established by other methodologies cannot be successfully reproduced in the Y2H assay and must therefore be considered as false-negative results. We failed, for example, to detect the interaction between RIT1/LZTR1 and KRAS/PI3Kα, which have been previously identified by biochemical techniques (42, 43). In our experience, ∼50% of PPIs that we tested worked in the Y2H system and were therefore amenable to DoMY-Seq. We recommend testing the binary interactions in a simple 1:1 test assay before undertaking DoMY-Seq. The chances of success are increased when testing the interaction pairs in different orientations and with both N- and C-terminal fusions of the DNA binding and activation domains. The recent release of the Human Reference Interactome’s ∼53,000 interaction pairs provide a convenient starting point for the design of many interaction pairs (44).

DoMY-Seq is the first methodology for the high-resolution characterization of protein–protein interaction sites in an *in vivo* context. While the concept of domain mapping by overlapping fragments has been introduced previously (44), DoMY-Seq provides maximal resolution with saturating numbers of fragments. Besides the identification of core binding domains as “plateaus,” the slopes that flank the core binding sequences may provide some unique insight into dynamic aspects of PPIs *in vivo*. Indeed, we find a minor enrichment in the CRD domain of CRAF, possibly reflecting a secondary binding or extended binding site of KRAS that was previously suggested by others (18, 45). Moreover, the shape of the peaks for MEK1 binding in CRAF shows preferential binding or potential additional binding sites toward the N terminus and away from the kinase domain. Hypotheses for preferential binding and minor binding sites can be further tested by altering sequence motifs in these flanking regions with site-directed mutagenesis, followed by analysis with DoMY-Seq.

Yeast assays provide an advantage over mammalian cell–based assays because they allow for the effective screening of complex libraries (46). Large numbers of desired variants can be enriched by simple selective cell growth using auxotrophic markers in small cultures. On the other hand, when studying mammalian proteins, Y2H may not capture the proper biological context of the PPI, such as higher order structures involving multiple proteins and post-translational modifications that do not occur in yeast (9). In addition, probing PPIs by interference with drugs in Y2H assays is affected by yeast metabolism such as the efficient removal of chemicals by the yeast multidrug resistance mechanisms (47). In the future, it would be interesting to also adapt the DoMY-Seq assay in a mammalian screening system (such as mammalian two-hybrid system; M2H system) that relies on cell-based sorting using fluorescent proteins (*i.e.*, GFP) or other selection markers (48). CRISPR/Cas9–based genome editing technologies may allow for an easier and more efficient adaptation of binary interaction assays to mammalian cell systems (49). Hence, we believe that interaction domain mapping using DoMY-Seq is poised to become an important tool for gaining detailed insights into poorly characterized protein complex associations and cellular signaling mechanisms.

## Experimental procedures

### Y2H strains

Two yeast strains were used in this study for the GAL4 system. One was *Saccharomyces cerαevisiae* Y2HGold (*MAT***a***, trp1–901, leu2–3,112, ura3–52, his3-200, gal4Δ, gal80Δ,LYS2::Gal1*_*UAS*_*-Gal1*_*TATA*_*-His3, GAL2*_*UAS*_*-Gal2*_*TATA*_*-Ade2, URA3::MEL1*_*UAS*_*-Mel1*_*TATA*_*, AUR1-C MEL1*), and the other was Y187 (*MATα, ura3-52, his3-200, ade2-101, trp-901, leu2-3, 112, gal4Δ, gal80Δ, met-, URA3:: Gal1*_*UAS*_*-Gal*_*TATA*_*-LacZ, MEL1*), which were purchased from Clontech.

### Construction of the bait vectors

To construct the human KRAS (G12V) bait vector, the human KRAS (G12V) ORFs were PCR amplified using Q5 DNA polymerase (NEB, Ipswich, MA) from a plasmid encoding KRAS (G12V) cDNA (Addgene # 83169) and was cloned into EcoRI/BamHI–digested pGBKT7-BD (designated as pGBKT7-KRAS G12V) by Gibson assembly and verified by Sanger sequencing. A list of primers used in the study is available in Table S1. The same procedures were performed for the construction of all other bait variants of KRAS (Q61L, WT, G12C, G12D, G13D), MEK1, p53, and RIT1. Additional mutations were generated using site-directed mutagenesis using KRAS G12V in pGBKT7 as a template, including the variants T35A, E37G, C180S, C186S, and CCSS. Site-specific mutagenesis was performed using a modified QuickChange protocol as described in (50). Briefly, PCR was performed with SuperFi DNA polymerase and DpnI-mediated digestion of the parental plasmid. The digest was then transformed into *Escherichia coli* for nick repair and then isolated and verified by Sanger sequencing of the bait insert.

### Autoactivation testing and adjustment of 3-AT concentration

The pGBKT7-KRAS (G12V) and all other bait constructs were transformed into the Y2HGold yeast strain by the lithium acetate method (51). The transformants were spread onto synthetic defined (SD)–Trp dropout plates and incubated at 30 °C for 3 days. All bait constructs were tested for self-activation, which is the ability to activate growth in the absence of a prey construct. We did not find significant self-activation in our bait constructs when selecting the prey strains on SD/-His/-Trp. Hence, selection for screens was performed on standard triple dropout medium SD/-His/Leu/-Trp plate and quadruple dropout medium SD/-Ade/-His/-Leu/-Trp.

### Library construction of the CRAF fragments for screens

To generate the CRAF fragments, the plasmid expressing the CRAF cDNA (pcDNA3-Myc-CRAF) was fragmented by acoustic shearing with a Covaris LE instrument (Covaris, Woburn, MA) in the 200- to 1000-bp range (5 fractions at 200 bp, 350 bp, 500 bp, 700 bp, and 1 kb). The DNAs were cleaned up, size selected using ProNex beads (Promega), and buffer exchanged into 62.5 μl of Illumina RSB. Sixty microliters was converted to NGS libraries using the TruSEq Nano kit (Illumina protocol 1000000040813 v00) starting with the “Repair Ends and Select Library Size” and the Illumina TruSeq Single Index Adapters, set A, and amplified according to this protocol. Figure S1 shows details of the TruSeq fragment construction method for DoMYSeq. The 5’ TruSeq adaptor sequence is translated into the linker protein sequence HSFPTRRSSDL. The frame of the 5’ adaptor is chosen so that a CT overhang remains in the adaptor, which then always results in a leucine at the junction between the vector/adaptor sequence and the insert. In this frame, the 3' T in the adaptor is not the first base for insert, and hence, artificial stop codons are avoided. Different frames for the 3’ adaptor sequences result in different stop codons.

DNA libraries were amplified using overhang oligos annealing to the TruSeq primer binding sites at their 3’ ends. The 5’ overhangs were homologous to the Y2H vectors pGBKT7 (Clontech) and pGAD-HA, depending on the target for library transformation (Fig. S1, panels A and B, respectively). The yeast libraries were constructed by homologous recombination in yeast. To generate the prey strain, the 5 different sized CRAF fragments and MDM2 and RGL3 fragment libraries were cotransformed with linearized (EcoRI-BamHI) pGAD-HA plasmids to generate pGAD-CRAF fragments in Y187 strain and Y2HGold-pGBKT7-KRAS (G12V) strain by homologous recombination (52, 53). We aim for ∼1 million transformants or primary clones per library transformation, to ensure saturating amounts of fragments covering every nucleotide position ∼100x with fragment ends (assuming plasmid sizes of ∼8–10 kb) over the plasmid template. The transformants were collected 2 days after growth at 30 °C and frozen at −80 °C in aliquots. The same procedures were followed for all other libraries. For the reverse assays, fragments were cloned into EcoRI/BamHI linearized pGBKT7 vector.

### Y2H screening

Before the NGS assay, screening conditions were tested in small-scale pilot assays. Upon combination of single-bait genes with prey libraries, and single preys with bait libraries for the reverse assays by yeast mating, small aliquots of cells were diluted and plated targeting 1:10,000 to 1:100,000 reporter activation per diploid cells. Baits with different KRAS bait variants were tested in Y2H mating assays with CRAF fragment libraries and full-length CRAF in pGAD-HA vector. Starting with 1 million cells, mated yeast cells were spotted in 5-fold dilution assays and grown on triple dropout medium SD/-His/Leu/-Trp and quadruple dropout medium SD/-Ade/-His/-Leu/-Trp for 3 days.

To examine the protein domain mapping, the Y2HGold transformed with the bait vector was mated with the Y187 c-Raf fragments library at 30 °C overnight on yeast extract peptone with 2% dextrose. The mated cells were then checked and spread on the 150-mm triple dropout medium SD/-His/Leu/-Trp plates and quadruple dropout medium SD/-Ade/-His/-Leu/-Trp plates to be incubated for 2 days. The Y2HGold-pGBKT7-k-Ras(G12V) yeast strain cotransformed with c-Raf fragments in pGAD-HA plasmids was also spread on the 150-mm SD/-His/Leu/-Trp plates and SD/-Ade/-His/-Leu/-Trp plates to be incubated for 2 days. The positive colonies were collected into an SD/-Ade/-His/-Leu/-Trp/liquid medium with 25% glycerol. The cells with an absorbance of 0.15 at 600 nm (*i.e.*, 1 ml of a culture at A_600_ 0.15) were inoculated into 20 ml of SD/-His/Leu/-Trp liquid medium and SD/-Ade/-His/-Leu/-Trp liquid medium and then incubated until the value of absorbance at 600 nm reached between 1 and 2. The prey plasmids were extracted by Plasmid DNA Rapidprep Mini kit (Tribioscience) after digestion with Zymolyase 20T. Yeast cells from the selected pool were suspended in 20 mM NaOH and heated at 99 °C for 20 min. An aliquot of the suspension was used for PCR amplification of the plasmid insert. The PCR product was subjected to cycle sequencing from 2 directions to map the insert clone.

### Library preparation for NGS

After screening and yeast plasmid DNA isolation, the concentration of double-stranded DNA was determined by Quantifluor (Promega) in comparison with lambda DNA standards. At this point, the library inserts do neither have Illumina indexes nor sequences required for annealing to the flow cell. Indexing primers from Paragon Genomics anneal at their 3’ end to the TruSeq sequencing primer site still in the two-hybrid library inserts and reintroduce indexes and the necessary P5 and P7 sequences. Fifteen nanograms of dsDNA was amplified with combinatorial indexing primers from Paragon Genomics in 50-μl reactions with 2 μl of i5 Indexed PCR Primer (Paragon Genomics), 2 μl of i7 Indexed PCR Primer (Paragon Genomics), 1 μl of P5, P7 primers mix (Illumina PPC), and 20 μl of EPM (Enhanced PCR Mix, Illumina) brought up to a final volume of 50 μl with PCR-grade water. Samples were amplified, according to the TruSeq Nano Reference Guide (Illumina # 1000000040135 v00), in an MJ Thermocycler with the heated lid set at 100 °C for 95 °C for 3 min, followed by eight cycles of 98 °C for 20 s, 60 °C for 15 s, 72 °C for 30 s, then 72 °C for 5 min, and hold at 4 °C. SPRI cleanup was adjusted for the target insert size. Size distributions and concentrations were verified by analysis on Bioanalyzer DNA7500 chips. Purified samples were then pooled and run on an Illumina MiSeq flow cell with 2 × 150-bp paired-end reads in either a full run or a MiSeq nano (MiSeq Reagent Kit v2). We found that ∼100,000 paired-end reads per mapping experiment are sufficient to characterize a binding site at nucleotide or amino acid resolution.

### Bioinformatics analysis of mapping

Raw Illumina read data were checked for quality with fastQC program (https://www.bioinformatics.babraham.ac.uk/projects/fastqc/. Accessed November 2019). Raw data were aligned to plasmid sequences from which the fragment libraries were generated using subread (http://bioinf.wehi.edu.au/subread/. Accessed November 2019). Custom scripts written in Perl and R were used to decide the ORF for each pair of reads relative to the start codon position of the inserted protein in the plasmid. Those pairs that map to the inserted protein region are from the correct frame and were extracted from the bam files in the previous step using the picard tool (https://github.com/broadinstitute/picard. Accessed November 2019).

The properly paired in-frame reads in each CDS sequence were further selected, and unique reads were selected using samtools (http://www.htslib.org/. Accessed November 2019). High-resolution interaction mapping data were generated from the unique paired reads: we assigned a reading frame to each pair and only took the pairs which are in the ORF with Raf1 CDS (frame 2 in this case). We consider not only reads within the CDS but also reads from the whole plasmid so we can see enrichment in CDS vs outside CDS. Negative frame means reverse strand. Construct design for in-frame translations are shown in Fig. S1.

We created coverage files by PE fragments in bigwig format from bam files of three types. All reads were all mapped reads, paired-end (PE), and singletons (only one read from the pair is mapped). UniquePE: singletons are discarded, and duplicate PE reads (those with the same start and end location) are removed to keep only one unique pair. InFrame: the UniquePE reads that are in frame with CDS. Coverage files were created in bigwig format using deepTools (https://deeptools.readthedocs.io/en/develop/index.html. Accessed November 2019). The coverage files are visualized using Integrated Genome Viewer (https://software.broadinstitute.org/software/igv/. Accessed November 2019).

Self-activating fragments were identified by analysis of the reads aligning to vectors using the 9-aa transactivation domain prediction tool (https://www.med.muni.cz/9aaTAD/index.php. Accessed November 2019).

### Visualization of BRAF/MEK crystal structure

To generate the snapshots of the BRAF/MEK1–binding interface, we used the crystal structure deposited at the Protein Data Bank (PDB ID: 4MNE), which was previously described (21). PyMol was used to visualize the structure and color the different domains and amino acids.

## Data availability

Primer sequences can be found in this manuscript as Table S1. Sequencing and Integrated Genome Viewer files can be requested to Bernhard P. Suter (suter@nextinteractions.com).

## Conflict of interest

P. C. is a cofounder and advisory board member of Venthera. F. M. is a consultant for Aduro Biotech, Amgen, Daiichi, Ideaya Biosciences, Kura Oncology, Leidos Biomedical Research, PellePharm, Pfizer, PMV Pharma, Portola Pharmaceuticals, and Quanta Therapeutics. He has received research grants from Daiichi and Gilead Sciences and is a consultant for and cofounder of BridgeBio Pharma, DNAtrix, Olema Pharmaceuticals, and Quartz. A. H.-M., Y. K., and B. P. S. are employees and stakeholders of Next Interactions.

## References

[1] Bonetta L. (2010). Protein-protein interactions: interactome under construction. Nature.

[2] Kiel C., Beltrao P., Serrano L. (2008). Analyzing protein interaction networks using structural information. Annu. Rev. Biochem..

[3] Yu H., Tardivo L., Tam S., Weiner E., Gebreab F., Fan C., Svrzikapa N., Hirozane-Kishikawa T., Rietman E., Yang X., Sahalie J., Salehi-Ashtiani K., Hao T., Cusick M.E., Hill D.E. (2011). Next-generation sequencing to generate interactome datasets. Nat. Methods.

[4] Ryan D.P., Matthews J.M. (2005). Protein-protein interactions in human disease. Curr. Opin. Struct. Biol..

[5] Simanshu D.K., Nissley D.V., McCormick F. (2017). RAS proteins and their regulators in human disease. Cell.

[6] Modell A.E., Blosser S.L., Arora P.S. (2016). Systematic targeting of protein-protein interactions. Trends Pharmacol. Sci..

[7] Miura K. (2018). An overview of current methods to confirm protein-protein interactions. Protein Pept. Lett..

[8] Xing S., Wallmeroth N., Berendzen K.W., Grefen C. (2016). Techniques for the analysis of protein-protein interactions *in vivo*. Plant Physiol..

[9] Mehla J., Caufield J.H., Uetz P. (2015). The yeast two-hybrid system: a tool for mapping protein-protein interactions. Cold Spring Harb. Protoc..

[10] Brückner A., Polge C., Lentze N., Auerbach D., Schlattner U. (2009). Yeast two-hybrid, a powerful tool for systems biology. Int. J. Mol. Sci..

[11] Vojtek A.B., Hollenberg S.M., Cooper J.A. (1993). Mammalian Ras interacts directly with the serine/threonine kinase Raf. Cell.

[12] Scheffler J.E., Waugh D.S., Bekesi E., Kiefer S.E., LoSardo J.E., Neri A., Prinzo K.M., Tsao K.L., Wegrzynski B., Emerson S.D. (1994). Characterization of a 78-residue fragment of c-Raf-1 that comprises a minimal binding domain for the interaction with Ras-GTP. J. Biol. Chem..

[13] Gibbs J.B., Schaber M.D., Marshall M.S., Scolnick E.M., Sigal I.S. (1987). Identification of guanine nucleotides bound to ras-encoded proteins in growing yeast cells. J. Biol. Chem..

[14] White M.A., Nicolette C., Minden A., Polverino A., Aelst L.V., Karin M., Wigler M.H. (1995). Multiple ras functions can contribute to mammalian cell transformation. Cell.

[15] Der C.J., Finkel T., Cooper G.M. (1986). Biological and biochemical properties of human rasH genes mutated at codon 61. Cell.

[16] Lin D.T.S., Davis N.G., Conibear E. (2017). Targeting the Ras palmitoylation/depalmitoylation cycle in cancer. Biochem. Soc. Trans..

[17] Khazak V., Golemis E.A., Weber L. (2005). Development of a yeast two-hybrid screen for selection of human Ras-Raf protein interaction inhibitors. Methods Mol. Biol..

[18] Winkler D.G., Cutler R.E., Drugan J.K., Campbell S., Morrison D.K., Cooper J.A. (1998). Identification of residues in the cysteine-rich domain of Raf-1 that control Ras binding and Raf-1 activity. J. Biol. Chem..

[19] Lavoie H., Therrien M. (2015). Regulation of RAF protein kinases in ERK signalling. Nat. Rev. Mol. Cell Biol..

[20] Karoulia Z., Gavathiotis E., Poulikakos P.I. (2017). New perspectives for targeting RAF kinase in human cancer. Nat. Rev. Cancer.

[21] Haling J.R., Sudhamsu J., Yen I., Sideris S., Sandoval W., Phung W., Bravo B.J., Giannetti A.M., Peck A., Masselot A., Morales T., Smith D., Brandhuber B.J., Hymowitz S.G., Malek S. (2014). Structure of the BRAF-MEK complex reveals a kinase activity independent role for BRAF in MAPK signaling. Cancer Cell.

[22] Thevakumaran N., Lavoie H., Critton D.A., Tebben A., Marinier A., Sicheri F., Therrien M. (2015). Crystal structure of a BRAF kinase domain monomer explains basis for allosteric regulation. Nat. Struct. Mol. Biol..

[23] Karoulia Z., Wu Y., Ahmed T.A., Xin Q., Bollard J., Krepler C., Wu X., Zhang C., Bollag G., Herlyn M., Fagin J.A., Lujambio A., Gavathiotis E., Poulikakos P.I. (2016). An integrated model of RAF inhibitor action predicts inhibitor activity against oncogenic BRAF signaling. Cancer Cell.

[24] Park E., Rawson S., Li K., Kim B.-W., Ficarro S.B., Pino G.G.-D., Sharif H., Marto J.A., Jeon H., Eck M.J. (2019). Architecture of autoinhibited and active BRAF–MEK1–14-3-3 complexes. Nature.

[25] Aoki Y., Niihori T., Banjo T., Okamoto N., Mizuno S., Kurosawa K., Ogata T., Takada F., Yano M., Ando T., Hoshika T., Barnett C., Ohashi H., Kawame H., Hasegawa T. (2013). Gain-of-function mutations in RIT1 cause Noonan syndrome, a RAS/MAPK pathway syndrome. Am. J. Hum. Genet..

[26] Shao H., Andres D.A. (2000). A novel RalGEF-like protein, RGL3, as a candidate effector for rit and Ras. J. Biol. Chem..

[27] Shi G.-X., Andres D.A. (2005). Rit contributes to nerve growth factor-induced neuronal differentiation via activation of B-Raf-extracellular signal-regulated kinase and p38 mitogen-activated protein kinase cascades. Mol. Cell. Biol..

[28] Hafner A., Bulyk M.L., Jambhekar A., Lahav G. (2019). The multiple mechanisms that regulate p53 activity and cell fate. Nat. Rev. Mol. Cell Biol..

[29] Vassilev L.T., Vu B.T., Graves B., Carvajal D., Podlaski F., Filipovic Z., Kong N., Kammlott U., Lukacs C., Klein C., Fotouhi N., Liu E.A. (2004). *In vivo* activation of the p53 pathway by small-molecule antagonists of MDM2. Science.

[30] Li Q., Lozano G. (2013). Molecular pathways: targeting Mdm2 and Mdm4 in cancer therapy. Clin. Cancer Res..

[31] Wong J.H., Alfatah M., Sin M.F., Sim H.M., Verma C.S., Lane D.P., Arumugam P. (2017). A yeast two-hybrid system for the screening and characterization of small-molecule inhibitors of protein–protein interactions identifies a novel putative Mdm2-binding site in p53. BMC Biol..

[32] Chen J., Marechal V., Levine A.J. (1993). Mapping of the p53 and mdm-2 interaction domains. Mol. Cell. Biol..

[33] Kussie P.H., Gorina S., Marechal V., Elenbaas B., Moreau J., Levine A.J., Pavletich N.P. (1996). Structure of the MDM2 oncoprotein bound to the p53 tumor suppressor transactivation domain. Science.

[34] Wallace M., Worrall E., Pettersson S., Hupp T.R., Ball K.L. (2006). Dual-site regulation of MDM2 E3-ubiquitin ligase activity. Mol. Cell..

[35] Poyurovsky M.V., Katz C., Laptenko O., Beckerman R., Lokshin M., Ahn J., Byeon I.-J.L., Gabizon R., Mattia M., Zupnick A., Brown L.M., Friedler A., Prives C. (2010). The C terminus of p53 binds the N-terminal domain of MDM2. Nat. Struct. Mol. Biol..

[36] Ma J., Ptashne M. (1987). A new class of yeast transcriptional activators. Cell.

[37] Piskacek M., Havelka M., Rezacova M., Knight A. (2016). The 9aaTAD transactivation domains: from Gal4 to p53. PLoS One.

[38] Murphy E. (1985). Nucleotide sequence of a spectinomycin adenyltransferase AAD(9) determinant from Staphylococcus aureus and its relationship to AAD(3") (9). Mol. Gen. Genet..

[39] Erffelinck M.-L., Ribeiro B., Perassolo M., Pauwels L., Pollier J., Storme V., Goossens A. (2018). A user-friendly platform for yeast two-hybrid library screening using next generation sequencing. PLoS One.

[40] Trigg S.A., Garza R.M., MacWilliams A., Nery J.R., Bartlett A., Castanon R., Goubil A., Feeney J., O’Malley R., Huang S.C., Zhang Z.Z., Galli M., Ecker J.R. (2017). CrY2H-seq: a massively multiplexed assay for deep-coverage interactome mapping. Nat. Methods.

[41] Starita L.M., Young D.L., Islam M., Kitzman J.O., Gullingsrud J., Hause R.J., Fowler D.M., Parvin J.D., Shendure J., Fields S. (2015). Massively parallel functional analysis of BRCA1 RING domain variants. Genetics.

[42] Castel P., Cheng A., Cuevas-Navarro A., Everman D.B., Papageorge A.G., Simanshu D.K., Tankka A., Galeas J., Urisman A., McCormick F. (2019). RIT1 oncoproteins escape LZTR1-mediated proteolysis. Science.

[43] Rodriguez-Viciana P., Warne P.H., Dhand R., Vanhaesebroeck B., Gout I., Fry M.J., Waterfield M.D., Downward J. (1994). Phosphatidylinositol-3-OH kinase direct target of Ras. Nature.

[44] Luck K., Kim D.-K., Lambourne L., Spirohn K., Begg B.E., Bian W., Brignall R., Cafarelli T., Campos-Laborie F.J., Charloteaux B., Choi D., Cote A.G., Daley M., Deimling S., Desbuleux A. (2019). A reference map of the human protein interactome. Syst. Biol..

[45] Mott H.R., Carpenter J.W., Zhong S., Ghosh S., Bell R.M., Campbell S.L. (1996). The solution structure of the Raf-1 cysteine-rich domain: a novel Ras and phospholipid binding site. Proc. Natl. Acad. Sci. U. S. A..

[46] Suter B., Zhang X., Pesce C.G., Mendelsohn A.R., Dinesh-Kumar S.P., Mao J.-H. (2015). Next-generation sequencing for binary protein–protein interactions. Front. Genet..

[47] Prasad R., Goffeau A. (2012). Yeast ATP-binding cassette transporters conferring multidrug resistance. Annu. Rev. Microbiol..

[48] He R., Li X. (2008). Mammalian two-hybrid assay for detecting protein-protein interactions *in vivo*. Methods Mol. Biol..

[49] Pickar-Oliver A., Gersbach C.A. (2019). The next generation of CRISPR–Cas technologies and applications. Nat. Rev. Mol. Cell Biol..

[50] Xia Y., Chu W., Qi Q., Xun L. (2015). New insights into the QuikChangeTM process guide the use of Phusion DNA polymerase for site-directed mutagenesis. Nucleic Acids Res..

[51] Gietz R.D., Schiestl R.H. (2007). Large-scale high-efficiency yeast transformation using the LiAc/SS carrier DNA/PEG method. Nat. Protoc..

[52] Gietz R.D., Schiestl R.H. (2007). High-efficiency yeast transformation using the LiAc/SS carrier DNA/PEG method. Nat. Protoc..

[53] Benatuil L., Perez J.M., Belk J., Hsieh C.-M. (2010). An improved yeast transformation method for the generation of very large human antibody libraries. Protein Eng. Des. Sel..

