# Supplementary material for: DoMY-Seq: A yeast two-hybrid–based technique for precision mapping of protein–protein interaction motifs
Source: J Biol Chem. 2020 Nov 23;296:100023. doi: 10.1074/jbc.RA120.014284 (PMC7949039; doi:10.1074/jbc.RA120.014284)
Supplement: Supplementary Figures and Tables [file mmc1.pdf]

Supporting Information

DoMY-Seq: A yeast two-hybrid-based technique for precision mapping of protein-protein interaction motifs

Pau Castel, Ann Holtz-Morris, Yongwon Kwon, Bernhard Suter, Frank McCormick

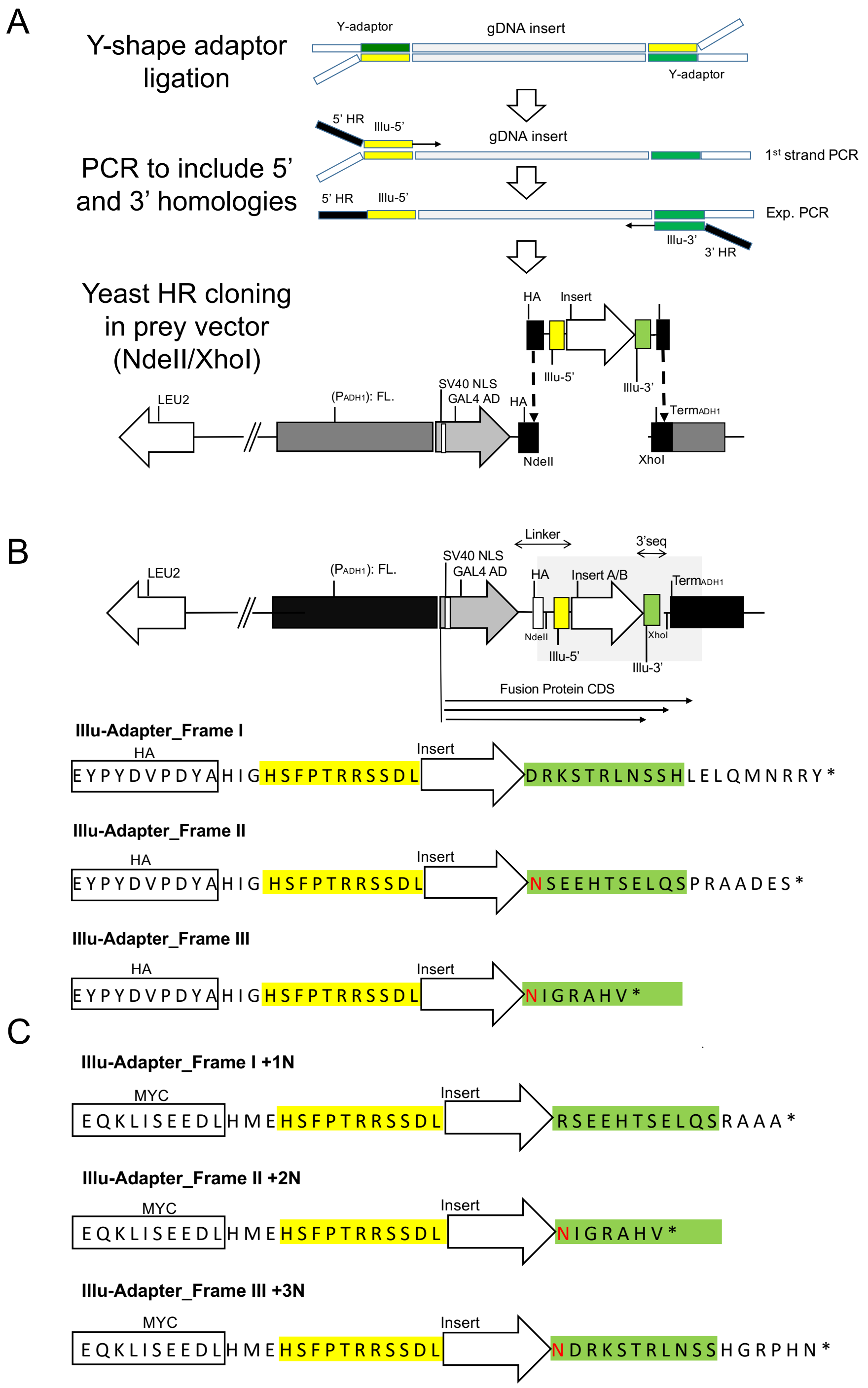

Figure S1. Overview library construction. (A) Experimental workflow for cloning library fragments for DoMY-Seq. DNA fragments are ligated with Y-adaptors, PCR amplified to introduce homology arms, and recombined into target vectors between NdeII and XhoI restriction enzyme sites. 50% of the fragments result in the right orientation. (B) Schematic overview of the different frames that are obtained in the prey vector (pGAD. While 33% of the insert are at the right frame, only half of these will be also at the right orientation. (C) Same as B for the bait vector (pGBKT7).

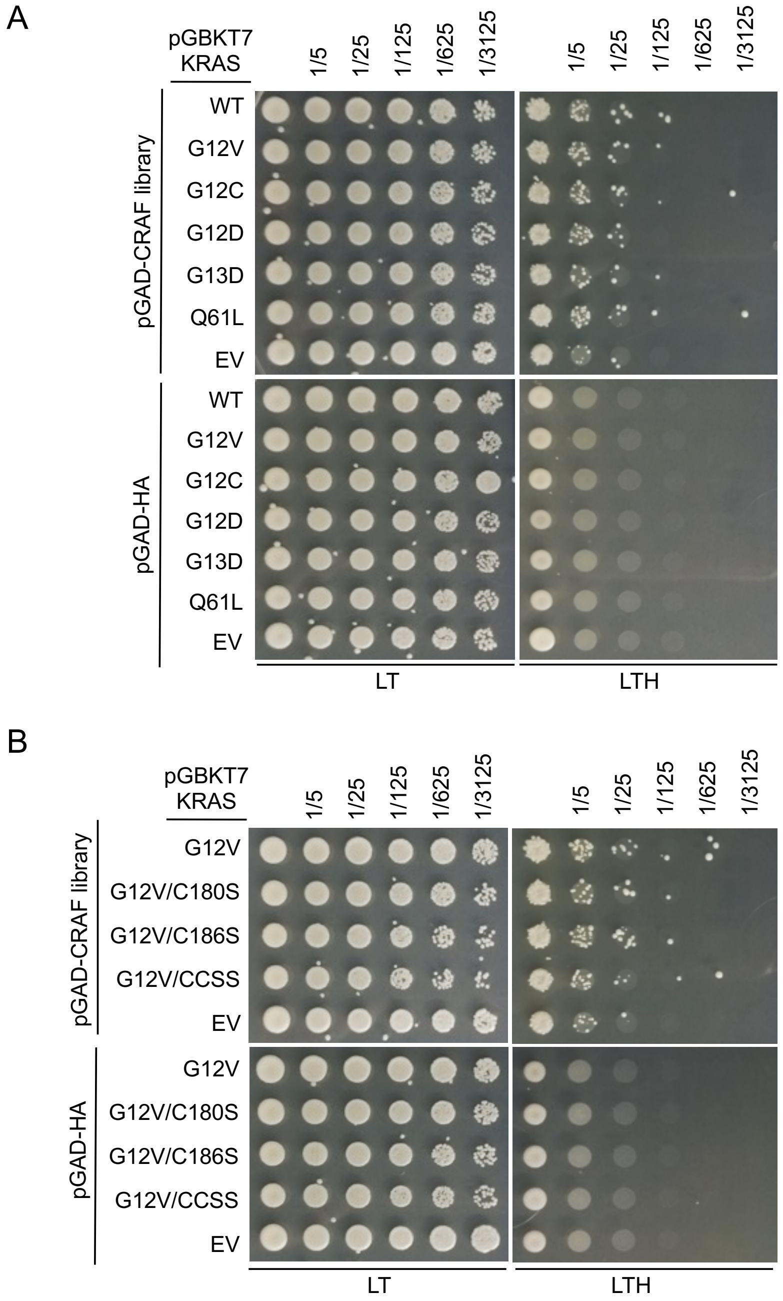

Figure S2. Optimization of the KRAS bait. (A) Colony growth assay using different KRAS oncogenic mutants in the bait vector pGBKT7 and mated to yeast containing the prey CRAF library in the pGAD vector. Colonies were incubated for 48 hours in basal media (LT) or dropout media (LTH). Five-fold dilutions are shown. (B) Colony growth assays as described in A using the C-terminal mutants that prevent lipidation of KRAS.

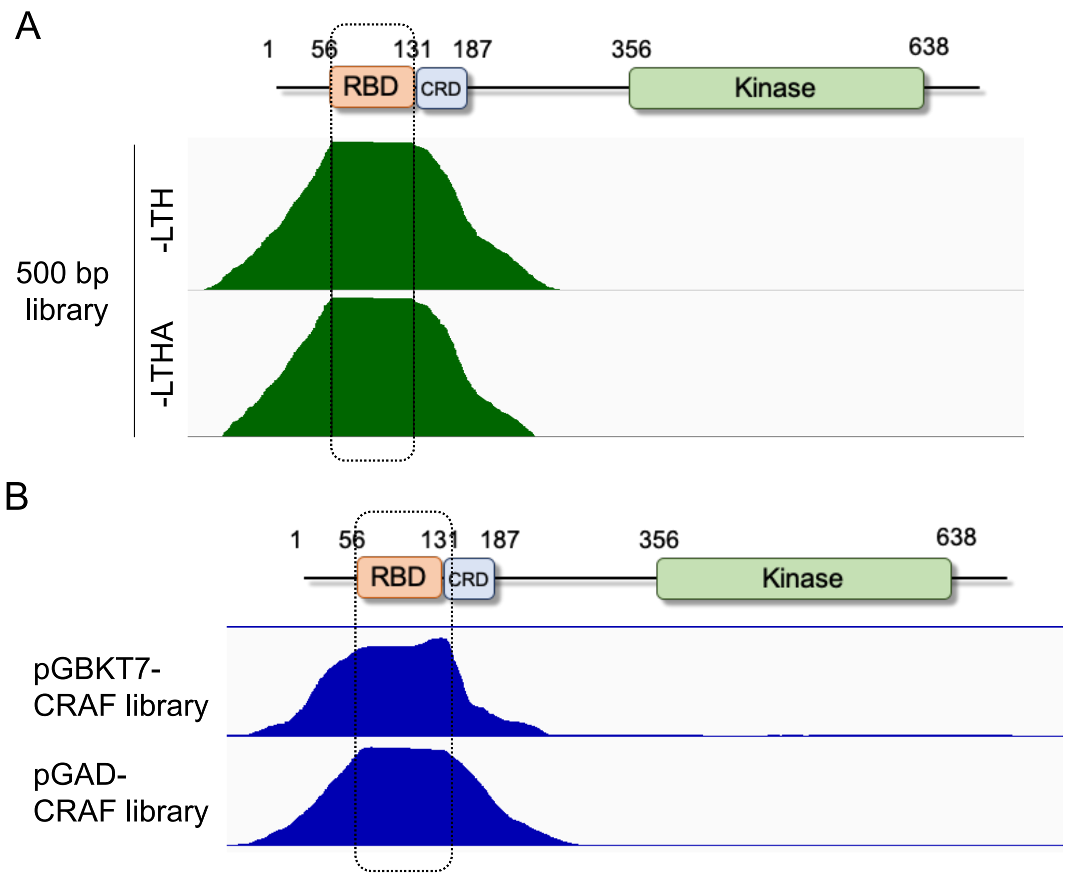

Figure S3. CRAF library cloned in the reversed orientation. (A) A library of longer CRAF fragments (~500 bp) was used for DoMY-Seq and revealed a similar KRAS-interacting motif when selected in dropout media -LTH or -LTHA. (B) The library of CRAF fragments was cloned in the pGAD vector instead of the pGBKT7 vector and assayed for binding to KRAS using DoMY-Seq. Although the binding motif obtained was similar in both assay orientations, the background reads were higher in the pGBKT7-library orientation.

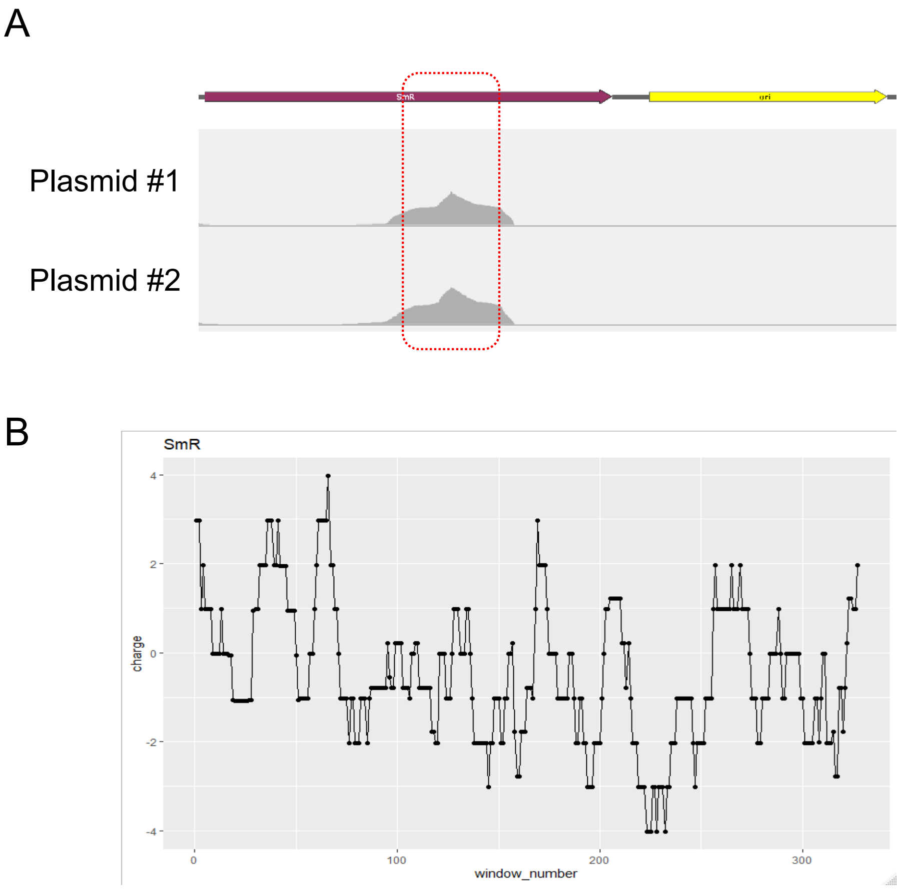

Figure S4. Acidic sequence in the Spectinomycin resistance gene. (A) Different plasmids used to generate libraries for DoMY-Seq in the bait (pGBKT7) orientation contained the Spectinomycin resistance gene (SmR). This sequence exhibited transactivation activity in our assay as shown in the IGV snapshots. Other regulatory sequences commonly found in plasmids (e.g. ori) were not found to contain such sequences. (B) A custom R script can be run to determine the charge of specific aa in a gene. Note the negatively charged sequence around amino acid position 225. The position of this sequence matches the transactivation region seen in A. Such transactivating regions (9aa TAD) are characterized by the presence of acidic, aromatic, and hydrophobic amino acids.

Bait cloning

Primer name	Sequence
pGBKT7-KRAS-Fw	GAGGAGGACCTGCATATGGCCATGGAGGCCGAATTCATGCCAACTTTGTACAAAAAAGTT
pGBKT7-KRAS-Rv	ATGCTAGTTATGCGGCCGCTGCAGGTCGACGGATCCTTACATTATAATGCATTTTTTAATTTTCACACAG
pLexA-RIT1-Fw	GTTGGGGTTATTCGCAACGGCGACTGGCTGGAATTCATGGATTCTGGAACTCGCCCAGTT
pLexA-RIT1-Rv	GGGCGAGCGAGTTGGTCGACCCGCGGCTGCAGGGTACCTCAAGTTACTGAATCTTTCTTC
pGBKT7-RIT1-Fw	TCAGAGGAGGACCTGCATATGGCCATGGAGGCCGAATTCATGGATTCTGGAACTCGCCCA
pGBKT7-RIT1-Rv	TGCTAGTTATGCGGCCGCTGCAGGTCGACGGATCCTTATCAAGTTACTGAATCTTTCTTC
pGAD-CRAF-Rv	GTATCTACGATTCATCTGCAGCTCGAGCTCGATGGATCCTTAGAAGACAGGCAGCCTCGG
pGAD-CRAF-Fw	CCAGATTACGCTCATATGGCCATGGAGGCCGAATTCATGCCAACTTTGTACAAAAAAGTT
pGBKT7-MEK1-Fw	CAGAGGAGGACCTGCATATGGCCATGGAGGCCGAATTCATGCCCAAGAAGAAGCCGACGC
pGBKT7-MEK1-Rv	TATGCTAGTTATGCGGCCGCTGCAGGTCGACGGATCCTTAGACGCCAGCAGCATGGGTTG
pGAD-P53-Fw	ACCAGATTACGCTCATATGAACATGGAGGCCAGTGAATTCATGGAGGAGCCGCAGTCAGA
pGAD-P53-Rv	GTATCTACGATTCATCTGCAGCTCGAGCTCGATGGATCCTCAGTCTGAGTCAGGCCCTTC

Mutagenesis

Primer name	Sequence
KRAS T35A Fw	GAATATGATCCAGCAATAGAGGATTCCTACAGG
KRAS T35A Rv	TCCTCTATTGCTGGATCATATTCGTCCACAAAATG
KRAS E37G Fw	CCAACAATAGGGGATTCCTACAGGAAGCAAG
KRAS E37G Rv	GTAGGAATCCCCTATTGTTGGATCATATTCG
KRAS C186S Fw	GAAAATTAAAAAAAGCATTATAATGTAAGGATCC
KRAS C186S Rv	CATTATAATGCTTTTTTTAATTTTCACACAGCCAGG
KRAS C180S Fw	GACTCCTGGCAGTGTGAAAATTAAAAAATGC
KRAS C180S Rv	ATTTTCACACTGCCAGGAGTCTTTTCTTCTTTG
KRAS CCSS Fw	GACTCCTGGCAGTGTGAAAATTAAAAAAAGCATTATAATGTAAGGATCC
KRAS CCSS Rv	CATTATAATGCTTTTTTTAATTTTCACACTGCCAGGAGTCTTTTCTTCTTTG

AdaptorHR

Primer name	Sequence
Illu5'_pGAD-AdaptorHR	CATGGAGTACCCATACGACGTACCAGATTACGCTCATATGGAACACTCTTTCCCTACACGACGCTCTTCCGATCT
Illu3'_pGAD-AdaptorHR	TGCGGGGTTTTTCAGTATCTACGATTCATCTGCAGCTCGAGGTGACTGGAGTTCAGACGTGTGCTCTTCCGATCT
Illu5'_pGBKT7_AdaptorHR	GAGGAGCAGAAGCTGATCTCAGAGGAGGACCTGCATATGGAACACTCTTTCCCTACACGACGCTCTTCCGATCT
Illu3'_pGBKT7_AdaptorHR	GAGGCCCCAAGGGGTTATGCTAGTTATGCGGCCGCGTGACTGGAGTTCAGACGTGTGCTCTTCCGATCT

Universal adapters (from Illumina)

Primer name	Sequence
TruSeq Universal Adapter	AATGATACGGCGACCACCGAGATCTACACTCTTTCCCTACACGACGCTCTTCCGATCT
TruSeq Adapter, Index 2	GATCGGAAGAGCACACGTCTGAACTCCAGTCACCGATGTATCTCGTATGCCGTCTTCTGCTTG
TruSeq Adapter, Index 4	GATCGGAAGAGCACACGTCTGAACTCCAGTCACTGACCAATCTCGTATGCCGTCTTCTGCTTG
TruSeq Adapter, Index 5	GATCGGAAGAGCACACGTCTGAACTCCAGTCACACAGTGATCTCGTATGCCGTCTTCTGCTTG
TruSeq Adapter, Index 6	GATCGGAAGAGCACACGTCTGAACTCCAGTCACGCCAATATCTCGTATGCCGTCTTCTGCTTG
TruSeq Adapter, Index 7	GATCGGAAGAGCACACGTCTGAACTCCAGTCACCAGATCATCTCGTATGCCGTCTTCTGCTTG
TruSeq Adapter, Index 12	GATCGGAAGAGCACACGTCTGAACTCCAGTCACCTTGTAATCTCGTATGCCGTCTTCTGCTTG
TruSeq Adapter, Index 15	GATCGGAAGAGCACACGTCTGAACTCCAGTCACATGTCAGAATCTCGTATGCCGTCTTCTGCTTG
TruSeq Adapter, Index 18	GATCGGAAGAGCACACGTCTGAACTCCAGTCACGTCCGCACATCTCGTATGCCGTCTTCTGCTTG

Indexing PCR primers (Paragon Genomics SKU 716005)

Primer name	Sequence
A501	AATGATACGGCGACCACCGAGATCTACACTGAACCTTACACTCTTTCCCTACACGACGCTCTTCCGATCT
A502	AATGATACGGCGACCACCGAGATCTACACTGCTAAGTACACTCTTTCCCTACACGACGCTCTTCCGATCT
A503	AATGATACGGCGACCACCGAGATCTACACTGTTCTCTACACTCTTTCCCTACACGACGCTCTTCCGATCT
A504	AATGATACGGCGACCACCGAGATCTACACTAAGACACACACTCTTTCCCTACACGACGCTCTTCCGATCT
A701	CAAGCAGAAGACGGCATACGAGATGTCGTGATGTGACTGGAGTTCAGACGTGTGCTCTTCCGATCT
A702	CAAGCAGAAGACGGCATACGAGATACCACTGTGTGACTGGAGTTCAGACGTGTGCTCTTCCGATCT
A703	CAAGCAGAAGACGGCATACGAGATTGGATCTGGTGACTGGAGTTCAGACGTGTGCTCTTCCGATCT
A704	CAAGCAGAAGACGGCATACGAGATCCGTTTGTGTGACTGGAGTTCAGACGTGTGCTCTTCCGATCT

Table S1. Primers used in this study.
